# The Ethics of Care Under Threat: When Protectors Become Perpetrators—Patterns of Abuse and Ethical Breaches by Physicians Accused of Sexual Misconduct in a Brazilian State

**DOI:** 10.1177/10778012251384624

**Published:** 2025-10-08

**Authors:** Renata Bittar Britto Arantes, Alanna Gomes da Silva, Mónica Maria Bessa Correia, Guilherme Augusto Veloso, Rui Manuel Lopes Nunes

**Affiliations:** 1 Faculty of Medicine, Bioethics Centre, 26705 University of Porto (UP), Porto, Portugal; 2 Department of Maternal and Child Nursing and Public Health, School of Nursing, 28114 Federal University of Minas Gerais (UFMG), Belo Horizonte, Brazil; 3 Faculty of Medicine, Department of Bioethics, 26705 University of Porto (UP), Porto, Portugal; 4 Department of Statistics, 28110 Fluminense Federal University (UFF), Rio de Janeiro, Brazil

**Keywords:** sexual harassment, physician–patient relationship, medical error, code of ethics, medical ethics

## Abstract

Sexual harassment perpetrated by physicians constitutes a critical form of institutional violence against women. This retrospective, cross-sectional study examined 39 ethical cases involving 37 male physicians judged by a Brazilian Ethics Tribunal between 2012 and 2022. Data were anonymized and analyzed using descriptive statistics and Barnard's test (*p* < .10). Most violations occurred within the field of Gynecology and Obstetrics, revealing breaches in the physician–patient relationship and infringements on human rights. Although many cases were dismissed for insufficient evidence, confirmed infractions resulted in severe sanctions. Findings underscore the urgency of ethical training and protective protocols to prevent harassment.

## Introduction

Allegations of sexual harassment have posed a persistent and significant challenge to adjudicating bodies across jurisdictions worldwide over the past few decades. This difficulty stems not only from the inherent complexity of such cases but also from the severe ethical, legal, and human implications they entail (Gabbard, 2014). By its very nature, sexual harassment constitutes a direct and intolerable violation of human dignity, fundamentally undermining the principle of autonomy—particularly the inalienable right of individuals to exert control over their own bodies and to freely determine matters concerning their physical and psychological integrity.

In this context, the body should be understood as the locus of subjectivity and emotion, where profound and complex human experiences—such as affection and aversion—are both housed and expressed. When this principle is violated, the individual is reduced to a mere object of domination, deprived of the freedom to exercise personal will, particularly in intimate or affective relationships (Gomes, 2007).

This underscores the centrality of the debate around the preservation of personal autonomy—conceived here as the individual's sovereign capacity to express inclinations, desires, and affections freely, within the private realm of human relations. Bodily self-determination, therefore, must not be regarded merely as an individual prerogative, but as a cornerstone of human dignity and ethical coexistence. The violation of such autonomy constitutes not only a personal affront but a broader attack on the moral order that sustains social relationships, especially those mediated by trust and emotional bonds. Abusive conduct thus transcends physical aggression, amounting to an assault on the victim's emotional autonomy, undermining the very pillars that uphold the freedom of affective and sexual expression (Gomes, 2007).

Such ethical transgressions become even more critical in the context of medical practice, where the power asymmetry between physician and patient is both institutionally recognized and socially legitimized. Within this caregiving relationship, patients—often in a state of physical or emotional vulnerability—consent to exposing their bodily and psychological intimacy to a health professional, driven by the legitimate expectation of receiving appropriate diagnostic and therapeutic care (Gonçalves, 2009). This act is an expression of trust, rooted in the premise that the ethical boundaries of the physician–patient relationship will be strictly upheld, and that the relationship will not deviate from the professional norms that define it (França, 2019).

Nevertheless, as highlighted by Bachmann et al. (2000), the practice of medicine inherently involves a high degree of physical and emotional proximity, which—under certain circumstances—can obscure the professional boundaries between those involved. These authors emphasize that the relational dynamics of medical care, marked by intimacy, trust, and power imbalance, mirror those found in family structures, rendering the physician–patient interaction particularly susceptible to boundary violations and abusive behaviors masked as therapeutic engagement.

Scholarly literature refers to this pathological dynamic as the Therapist–Patient Sexual Syndrome. Within such relationships, a deep, primitive, and quasi-infantile bond may develop, akin to that of a child with a parental figure. The physician, in this context, may be perceived as an omnipotent figure, imbued with almost magical qualities—idealized and simultaneously feared. When the therapist reinforces such transference and exploits the therapeutic relationship sexually—an unequivocally antitherapeutic act—the patient may feel psychologically trapped. On one hand, they seek union with the physician, not only for care but also for protection; on the other hand, akin to a child abused by a caregiver, they feel fear and a desire to escape. Patients may also experience guilt for engaging sexually, mistakenly believing they are in control of the situation. Regardless of whether the patient expresses physical attraction or engages in flirtatious behavior, the responsibility for any abuse lies solely with the healthcare professional (Feldman-Summers & Jones, 1984).

This complex emotional and interpersonal dynamic has been interpreted by scholars as analogous to incestuous abuse, marked by power imbalances, emotional dependency, and violations of the ethical boundaries that should govern therapeutic relationships (Council on Ethical and Judicial Affairs, American Medical Association, 1991; Limbert & Microys, 1991).

Sexual contact between physicians and patients encompasses any physical interaction intended to stimulate or satisfy the sexual desires of the physician, the patient, or both. Such conduct constitutes an ethical violation regardless of who initiates the sexualization of the relationship, which must, by definition, remain professional and grounded in mutual respect.

Sexual contact in these circumstances cannot be deemed consensual, as the patient lacks the ability to provide morally valid consent. The notion of consent is fundamentally compromised in these scenarios due to the inherent vulnerability and dependency of the patient. For consent to be legitimate, it must reflect a fully autonomous and intentional choice, based on a comprehensive, informed understanding, and made in an environment free from coercion, manipulation, or undue influence (Canadian Medical Association, 1994; Gauer et al., 2001).

Moreover, patients may struggle to recognize abusive behavior from professionals, especially as such conduct rarely begins with overt acts—such as vaginal or anal penetration—but typically escalates through subtle behaviors like unsolicited touching of intimate areas or sexually suggestive remarks. This insidious progression impairs the victim's ability to perceive the abuse in its early stages. Recognition often occurs only when the harassment intensifies, by which time the victim may experience intense feelings of guilt, shame, and fear, particularly in the face of anticipated disbelief due to a lack of prior complaints (Fávero et al., 2023).

Gender dynamics emerge sharply in this scenario. A significant body of research has shown that perpetrators in therapeutic contexts are predominantly male, while the victims are overwhelmingly female. This pattern is not incidental; rather, it is deeply rooted in social and cultural structures that normalize and perpetuate male dominance and the objectification of the female body. The physician–patient power asymmetry—already intensified by the symbolic authority of the healthcare professional—becomes even more critical when intersected by gender, wherein the female body remains a target of systemic control, silencing, and repeated violence (Gauer et al., 2014).

In light of this, it is important to understand the diverse psychopathological profiles associated with offending physicians. Some may present with psychotic disorders, often leading to less elaborate, lower-impact conduct, requiring pharmacological and psychotherapeutic intervention. Others exhibit antisocial traits and engage in systematically predatory behavior, frequently associated with early-life sexual victimization. These individuals tend to instrumentalize patients as objects for personal gratification, devoid of emotional bonds. There are also professionals with narcissistic traits who eroticize therapeutic interactions, blurring the boundaries of clinical practice. Finally, some offenders display masochistic traits, recognize the severity of their actions, and may self-report in search of help (Cataldo & Gauer, 2005).

Despite the severity of this phenomenon and its impact on both victims and professional ethics, there is a notable lack of empirical and theoretical studies addressing sexual violence perpetrated by healthcare professionals on an international scale. The academic literature remains nascent, both in conceptual development and in the systematic collection of data (Fávero et al., 2023). In Brazil, this gap is even more pronounced, constituting an epistemological void that hampers the development of public policies and the formulation of effective prevention and accountability strategies (Gauer et al., 2014).

Although academic output on the subject remains limited, it is imperative to recognize that existing legal and ethical frameworks—both in criminal law and medical ethics codes—clearly and unequivocally prohibit coercive sexual behavior, particularly when perpetrated by individuals in positions of authority or hierarchical superiority. The current edition of the Brazilian Code of Medical Ethics reinforces this stance, classifying any violation of human dignity or professional morality as an ethical infraction, regardless of the form or stated motivation of such behavior. However, the imposition of sanctions requires a case-by-case assessment, based on a technically sound, individualized analysis of each situation. As expected of any adjudicative body committed to justice and due process, such deliberations must be conducted with both sensitivity and rigor, ensuring that outcomes are fair and proportionate to the circumstances of each case (Conselho Federal de Medicina, 2018).

In these proceedings, Brazil's Regional Medical Councils are responsible for initiating psychiatric evaluations of accused physicians when there is reason to suspect a mental health disorder. This preliminary step, conducted by a medical board, precedes the ethical-professional inquiry and aims to determine the physician's capacity to practice. This procedure ensures that any assessment of accountability considers the mental health status of the professional, promoting a more balanced and just evaluation in accordance with Federal Medical Council guidelines (Conselho Federal de Medicina, 2022).

It is also relevant to highlight that many complaints reviewed by medical ethics councils originate from prior judicial proceedings. This can be attributed to victims’ understandable reluctance to relive the traumatic experience of abuse, often marked by fear, shame, or the desire to avoid public exposure and confrontation with the perpetrator. Given the private and intimate nature of most harassment episodes—typically occurring within the confines of the physician–patient interaction—such reports frequently lack robust evidentiary support, such as forensic documentation, imagery, or eyewitness accounts. In the absence of material evidence, the legal principle of *in dubio pro reo* may prevail, potentially precluding the imposition of disciplinary or criminal sanctions (Cohen et al., 2009; Mariani, 2021). Nevertheless, once the veracity of allegations is established, sexual harassment is classified as both a grave ethical violation and an aggravated criminal offense, especially in light of the patient's vulnerability—often associated with pain, suffering, or dependency—which contributes to their silence and hesitation to report, driven by a complex mix of fear, shame, and potential re-victimization (Gonçalves, 2009; Reling et al., 2018).

Nationally, one of the few studies on this issue has shown a marked concentration of complaints in Brazil's Southeast region, likely due to its higher density of medical professionals (Gomes, 2007). Within this landscape, the state of Minas Gerais stands out as home to the country's third-largest medical community, thus justifying its selection as the empirical field for the present investigation. In light of these considerations, this study seeks to address the academic literature gap by offering a detailed analysis of Professional Ethical Proceedings related to sexual harassment adjudicated by the Regional Medical Council of Minas Gerais (CRM-MG) between 2012 and 2022. Through an in-depth investigation of these cases, the study aims to expand understanding of the underlying dynamics of this form of violence, with a particular focus on power asymmetries and gendered structures of violence that pervade physician–patient relationships—frequently obstructing disclosure, investigation, and ultimately, perpetrator accountability.

## Methodology

### Study Design

This study employed a retrospective, cross-sectional, descriptive, and exploratory design, integrating both quantitative and qualitative data.

### Data Sources

Official data were provided by the Board of Directors of the Regional Medical Council of Minas Gerais (CRM-MG) following a formal request. The data were extracted from the institution's Case Processing Department database, after a filtering procedure and removal of sensitive information, in compliance with the Brazilian General Data Protection Law (Law No. 13.709/2018) (Brazil, 2018). As such, the dataset excluded any information that could lead to the identification of individuals.

### Settings and Participants

The dataset comprised 39 Professional Ethical Proceedings (PEPs) involving 37 physicians. The inclusion criteria encompassed all completed ethical proceedings judged between 2012 and 2022. Cases with incomplete data or that remained unresolved by the end of 2022 were excluded from the analysis.

### Variables

The sociodemographic variables related to the physicians included:

- Gender (male or female)
- Age group
- Years of medical practice
- Medical specialty (generalist or specialist)
- Legal nature of the undergraduate medical institution (public or private)
- State of the undergraduate institution (Minas Gerais or other Brazilian states)
- Nationality (Brazilian or foreign)

Contextual variables related to the professional ethical proceedings included:

- Gender of the victim (male or female)
- Source of complaint (CRM-MG or patients/family members)
- Location of the incident (urban capital or countryside)
- Legal nature of the institution where the incident occurred (public or private)
- Relevant chapters and articles of the Code of Medical Ethics cited and violated
- Determination of guilt (yes or no)
- Type of sanction imposed (confidential warning—A; confidential reprimand—B; public reprimand—C; temporary suspension of medical practice for 30 days—D; or permanent revocation of medical license—E)
- Appeal to the Federal Medical Council (CFM) (yes or no, including whether the sanction was upheld, reduced, or increased)

### Bias

For the two physicians identified as repeat defendants in cases of sexual misconduct, only the first proceeding of each individual was included in the analysis, in order to minimize potential sources of bias in the results.

### Statistical Methods

The behavior of the observed variables was analyzed using descriptive statistics. For qualitative variables, absolute and relative frequencies (percentages) were calculated. For quantitative variables, the arithmetic mean was used as a measure of central tendency.

Following this initial descriptive phase, a comprehensive analysis was conducted to examine associations between the physicians’ sociodemographic and procedural characteristics and the most frequently cited chapters of the Brazilian Code of Medical Ethics. The aim was to identify significant statistical correlations.

Subsequently, these variables were cross-referenced with the final determination of guilt in order to assess whether sociodemographic or procedural characteristics influenced the likelihood of a physician being found guilty. To examine associations between categorical variables, Barnard's exact test was applied, adopting a significance level of 10% (*p* < .10).

### Ethical Considerations

The doctoral project from which the present article is derived was subjected to rigorous review by the Ethics Committee of the University of Porto, which issued a favorable opinion on June 29, 2023, under protocol number 111/CEFMUP/2023.

## Results

A total of 39 Professional Ethical Proceedings (PEPs) involving 37 physicians were analyzed. All cases were adjudicated at first instance by the Regional Medical Council of Minas Gerais (CRM-MG) between January 2012 and December 2022. Among the 37 physicians judged, all were male (100%), with a mean age of 46 years, ranging from 24 to 70 years. It was observed that 25% were under 37 years old, 50% were under 42, and 75% were under 54. The most prevalent age group was 31−50 years (59.5%), followed by those over 50 (32.4%) and under 30 (8.1%) (Table 1).

**Table 1.** Demographic Variables Involved in the First Legal Action of Each Physician.

Sexual Harassment	*N*	%
*Gender*		
Male	37	100
Female	0	0
*Age group*		
≤30	3	8.1
31−50	22	59.5
≥50	12	32.4
*Years in medical practice*		
≤10	12	32.4
11−20	10	27
≥20	15	40.5
*Nationality*		
Brazilian	36	97.3
Foreign	1	2.7
*Legal status of medical degree*		
Private	16	43.2
Public	21	56.8
*Revalidation*		
No	36	97.3
Yes	1	2.7
*Specialty status*		
Specialist	22	59.5
General practitioner	15	40.5

*Source:* Author's own elaboration based on raw data from the Regional Medical Council of Minas Gerais.

The mean length of medical practice was 18.2 years. A quarter of the physicians had less than 9 years of experience, 50% had less than 13 years, and 75% had less than 27 years. The group with 20 or more years of professional practice was the most prevalent (Table 1).

Only 2.7% of the physicians were foreign-born, while 97.3% were Brazilian. Among Brazilian graduates, 56.8% earned their degrees from public institutions and 43.2% from private institutions (Table 1). The majority of these medical schools were located in Minas Gerais (70.3%), followed by Rio de Janeiro (18.9%), Espírito Santo (2.7%), Alagoas (2.7%), Pará (2.7%), and Amazonas (2.7%).

A greater prevalence of general practitioners (40.5%) was noted, followed by specialists in gynecology and obstetrics (16.2%), cardiology (8.1%), and orthopedics and traumatology (8.1%). The most frequently reported specialties were gynecology and obstetrics (27%), internal medicine (21.6%), cardiology (8.1%), and occupational medicine (8.1%) (Table 2).

**Table 2.** Specialties and Areas Involved in the First Legal Action of Each Physician.

	Accused (%)	Convicted (%)	Acquitted (%)
*Medical specialty*			
No registered medical specialty	15 (40.5)	9 (60)	6 (40)
Gynecology and obstetrics	6 (16.2)	2 (33.3)	4 (66.7)
Cardiology	3 (8.1)	1 (33.3)	2 (66.7)
Orthopedics and traumatology	3 (8.1)	0 (0)	3 (100)
General surgery	2 (5.4)	0 (0)	2 (100)
Internal medicine	2 (5.4)	1 (50)	1 (50)
Neurology	2 (5.4)	1 (50)	1 (50)
Pediatrics	2 (5.4)	1 (50)	1 (50)
Allergy and immunology	1 (2.7)	0 (0)	1 (100)
Dermatology	1 (2.7)	0 (0)	1 (100)
Endocrinology	1 (2.7)	1 (100)	0 (0)
Gastroenterology	1 (2.7)	0 (0)	1 (100)
Traffic medicine	1 (2.7)	0 (0)	1 (100)
Occupational medicine	1 (2.7)	0 (0)	1 (100)
Forensic medicine and medical Expertise	1 (2.7)	0 (0)	1 (100)
Neurosurgery	1 (2.7)	1 (100)	0 (0)
Nutrition	1 (2.7)	1 (100)	0 (0)
Psychiatry	1 (2.7)	1 (100)	0 (0)
*Reported area*			
Gynecology and obstetrics	10 (27)	4(40)	6 (60)
Internal medicine	8 (21.6)	5 (62.5)	3 (37.5)
Cardiology	3 (8.1)	1 (33)	2 (50)
Occupational medicine	3 (8.1)	1 (33)	2 (67)
General surgery	2 (5.4)	0 (0)	2 (100)
Neurology	2 (5.4)	1 (50)	1 (50)
Orthopedics and traumatology	2 (5.4)	0 (0)	2 (100)
Dermatology	1 (2.7)	0 (0)	1 (100)
Endocrinology	1 (2.7)	1 (100)	0 (0)
Endoscopy	1 (2.7)	0 (0)	1 (100)
Pediatrics	1 (2.7)	1 (100)	0 (0)
Psychiatry	1 (2.7)	1 (100)	0 (0)
Radiology and diagnostic imaging	1 (2.7)	0 (0)	1 (100)
No reported area	1 (2.7)	0 (0)	1 (100)

*Source:* Author's own elaboration based on raw data from the Regional Medical Council of Minas Gerais.

Among the victims in the analyzed cases, 92.8% were female and 7.2% were male. In two proceedings, the number of victims was unspecified, although both male and female victims were indicated. Regarding the complainant profile, most reports (59.5%) were initiated by CRM-MG, followed by complaints filed by patients or their family members (40.5%). As for the location of the incidents, the majority occurred in the interior regions of Minas Gerais (64.9%). With respect to the legal nature of the institutions where the misconduct occurred, private institutions accounted for the highest number of complaints (56.8%) (Table 3).

**Table 3.** Sex of the Victims, Profile of the Complainant, Location of the Complaint, Outcome of the Process, and Recidivism Involved in the First Legal Action of Each Physician.

Variable	*N*	%
*Gender of the victims*		
Female	64	92.8
Male	5	7.2
*Complainant*		
CRMMG ex officio	22	59.5
Individual	15	40.5
*Location of the complaint*		
Capital	13	35.1
Interior	24	64.9
*Legal status of reporting* Location		
Public	16	43.2
Private	21	56.8
*Outcome of the process*		
Conviction	16	43.2
Acquittal	21	56.8
*Recidivism*		
Yes	2	5.4
No	35	94.6

*Source:* Author's own elaboration based on raw data from the Regional Medical Council of Minas Gerais.

The most frequently cited chapters of the Medical Ethics Code related to the cases involved human rights, the physician–patient relationship, and professional responsibility. Similarly, the most frequently violated chapters were those concerning patient and family relations, human rights, and professional responsibility (Annex 1).

The most cited articles of the Medical Ethics Code were Articles 38 (75.7%), 40 (64.9%), 30 (62.2%), and 23 (51.4%), while the most violated were Articles 38 (50%), 30 (50%), 40 (43.8%), and 23 (12.5%) (Table 4).

**Table 4.** Definition and Rate of Capitulation/Infraction of the Most Prevalent Articles Related to Sexual Harassment.

Articles	Definition—It is Prohibited for a Physician	Capitulations (37 Physicians)		Infractions (16 Physicians)	
		Quantity	%	Quantity	%
38	Disregard the modesty of any person under their professional care.	28	75.7	8	50.0
40	Exploit situations arising from the physician–patient relationship to gain physical, emotional, financial, or any other advantage.	24	64.9	7	43.8
30	To use their profession to corrupt morals, commit, or facilitate crimes.	23	62.2	8	50.0
23	To treat any human being without civility or consideration, to disrespect their dignity, or to discriminate against them in any way or under any pretext. Sole paragraph. The physician must maintain respect, consideration, and solidarity with their colleagues.	19	51.4	2	12.5

*Source:* Author's own elaboration based on raw data from the Regional Medical Council of Minas Gerais.

The overall acquittal rate was 56.8%, while the conviction rate was 43.2%. The most commonly applied sanctions included temporary suspension of professional activity for 30 days (penalty D: 16.2%), public censure (penalty C: 13.5%), revocation of licensure (penalty E: 8.1%), confidential warning and confidential censure (penalties A and B: 2.7% each).

Appeals to the Federal Council of Medicine (CFM) were filed in 21.6% of the cases, while the majority (78.4%) did not result in an appeal. Among the appealed cases, most decisions were upheld (62.5%), while 37.5% resulted in reduced penalties. No cases of increased penalties were observed. Recidivism was identified in two of the 37 physicians.

With regard to associations between sociodemographic or procedural variables and the most cited ethical chapters related to sexual harassment in the Medical Ethics Code, associations were found between specialty (generalist or specialist) and the chapter on the physician–patient relationship (*p = *.084), between the type of complainant (CRM-MG or patients/families) and the chapter on human rights (*p = *.095), and between the location of the offense (urban or rural) and the chapter on human rights (*p = *.074) (Table 5).

**Table 5.** Association between Demographic Variables, Complainant, Location of the Infraction, and the Chapters of Professional Responsibility, Human Rights, and the Doctor–Patient Relationship Involved in the First Legal Action of Each Physician.

Codified Articles									
		Human Rights				Physician–Patient Relationship			
Variable	Category	No (%)	Yes (%)	Total (%)	*p*	No (%)	Yes (%)	Total (%)	*p*
Age group	≤40 years	3 (50)	12 (38.7)	15 (40.5)	.444	4 (57.1)	11 (36.7)	15 (40.5)	.218
	>40 years	3 (50)	19 (61.3)	22 (59.5)		3 (42.9)	19 (63.3)	22 (59.5)	
Years in medical practice	≤15 years	3 (50)	16 (51.6)	19 (51.4)	.591	4 (57.1)	15 (50)	19 (51.4)	.551
	>15 years	3 (50)	15 (48.4)	18 (48.6)		3 (42.9)	15 (50)	18 (48.6)	
Legal status of medical degree	Private	3 (50)	13 (41.9)	16 (43.2)	.589	2 (28.6)	14 (46.7)	16 (43.2)	.293
	Public	3 (50)	18 (58.1)	21 (56.8)		5 (71.4)	16 (53.3)	21 (56.8)	
State where degree was obtained	Minas Gerais	4 (66.7)	22 (71)	26 (70.3)	.589	5 (71.4)	21 (70)	26 (70.3)	.556
	Other	2 (33.3)	9 (29)	11 (29.7)		2 (28.6)	9 (30)	11 (29.7)	
Complainant	CRMMG (ex officio)	2 (33.3)	20 (64.5)	22 (59.5)	.095	3 (42.9)	19 (63.3)	22 (59.5)	.218
	Patient	4 (66.7)	11 (35.5)	15 (40.5)		4 (57.1)	11 (36.7)	15 (40.5)	
Legal status of reporting location	Private	3 (50)	18 (58.1)	21 (56.8)	.589	5 (71.4)	16 (53.3)	21 (56.8)	.293
	Public	3 (50)	13 (41.9)	16 (43.2)		2 (28.6)	14 (46.7)	16 (43.2)	
Specialty status	Specialist	5 (83.3)	17 (54.8)	22 (59.5)	.156	2 (28.6)	20 (66.7)	22 (59.5)	.084
	General practitioner	1 (16.7)	14 (45.2)	15 (40.5)		5 (71.4)	10 (33.3)	15 (40.5)	
Location	Capital	4 (66.7)	9 (29)	13 (35.1)	.074	2 (28.6)	11 (36.7)	13 (35.1)	.551
	Countryside	2 (33.3)	22 (71)	24 (64.9)		5 (71.4)	19 (63.3)	24 (64.9)	

*Source:* Author's own elaboration based on raw data from the Regional Medical Council of Minas Gerais.

A statistically significant association was found between medical specialty (generalist or specialist) and the determination of culpability (yes or no) (*p = *.05) (Table 6).

**Table 6.** Association Between Demographic Variables, Complainant, Location of the Infraction, and Culpability Involved in the First Legal Action of Each Physician.

First Disciplinary Proceeding for Each Physician					
		Culpability Assessment			
Variable	Category	Not Guilty (%)	Guilty (%)	Total (%)	*p*
Age group	≤40 years	7 (33.3)	8 (50)	15 (40.5)	.213
	>40 years	14 (66.7)	8 (50)	22 (59.5)	
Years in medical practice	≤15 years	10 (47.6)	9 (56.2)	19 (51.4)	.325
	>15 years	11 (52.4)	7 (43.8)	18 (48.6)	
Legal status of medical degree	Private	10 (47.6)	6 (37.5)	16 (43.2)	.322
	Public	11 (52.4)	10 (62.5)	21 (56.8)	
State where degree was obtained	Minas Gerais	16 (76.2)	10 (62.5)	26 (70.3)	.213
	Other	5 (23.8)	6 (37.5)	11 (29.7)	
Complainant	CRMMG (ex officio)	14 (66.7)	8 (50)	22 (59.5)	.213
	Individual	7 (33.3)	8 (50)	15 (40.5)	
Legal status of infraction location	Private	11 (52.4)	10 (62.5)	21 (56.8)	.322
	Public	10 (47.6)	6 (37.5)	16 (43.2)	
Location of infraction	Capital	7 (33.3)	6 (37.5)	13 (35.1)	.429
	Countryside	14 (66.7)	10 (62.5)	24 (64.9)	
Specialization status	Specialist	15 (71.4)	7 (43.8)	22 (59.5)	.054
	General practitioner	6 (28.6)	9 (56.2)	15 (40.5)	

*Source:* Author's own elaboration based on raw data from the Regional Medical Council of Minas Gerais.

## Discussion

Despite encompassing a ten-year period and focusing on Minas Gerais—the Brazilian state with the third-largest number of physicians — this study identified only 39 Professional Ethical Proceedings involving 37 physicians. This relatively small number of cases suggests substantial underreporting, as previously pointed out by Fávero et al. (2023). The low number of formal complaints reflects the significant challenges many victims face in reporting traumatic experiences.

A marked predominance of male physicians among those accused of sexual harassment was observed, consistent with previous findings in the literature on inappropriate conduct in medical practice (Cohen et al., 2009; Gartrell et al., 1986; Gomes, 2007; Kardener et al., 1973; Pope & Bouhoutsos, 1986; Sansone & Sansone, 2009; Schoener et al., 1989). Gomes (2007) reported that 94.8% of accused physicians were male, while Cohen et al. (2009) found that 96.7% were men. Similarly, Sansone and Sansone (2009) indicated that over 85% of involved professionals were male, highlighting a significant gender disparity in these ethical violations.

Most accused physicians were between 31 and 50 years old, echoing findings by Pope and Bouhoutsos (1986). This age concentration may reflect social factors such as affective restructuring, family difficulties, marital instability, and stress related to professional overexertion in response to societal demands. While age analysis offers insights, it remains insufficient to fully explain the phenomenon of harassment, which must be contextualized within a broader matrix of personal, psychological, and professional factors (Gomes, 2007).

The most common professional experience among accused physicians was 20 years or more. This may suggest a link between advanced age and sexual misconduct, potentially associated with psychodynamic factors related to aging. The psychic structures of aging professionals, when confronted with existential insecurities, may influence the emergence of abusive behaviors, particularly when compounded by personality traits and stressors that lead to the displacement of personal frustrations into clinical interactions (Cohen et al., 2009).

Although more accused physicians graduated from public institutions, no significant correlation was identified between the type of medical school and sexual harassment behavior. Cohen et al. (2009) emphasized that such violations occur regardless of academic background, suggesting that interpersonal dynamics, power structures, and ethical maturity are more critical factors than academic pedigree.

The study showed a high concentration of accused physicians originating from Minas Gerais, Rio de Janeiro, and Espírito Santo, potentially reflecting regional sociocultural traits, levels of urbanization, and professional density. However, these patterns should not be generalized, as inappropriate behavior may occur across all regions, especially where certain practices are normalized.

General practitioners, followed by gynecologists and obstetricians, were most frequently implicated. It is plausible that generalists, who often work unsupervised and in diverse clinical contexts, may face more ethical challenges. While specialized training theoretically equips professionals with better technical and ethical competencies, further investigation is necessary to clarify this correlation.

Gynecology and obstetrics stood out as the most frequently reported specialties, corroborating previous findings (Cohen et al., 2009; Fávero et al., 2023; Gomes, 2007; Kardener et al., 1973; Sansone & Sansone, 2009). The intimate nature of gynecological procedures, combined with the inherent power imbalance in clinical encounters, creates a vulnerable environment susceptible to abuse. As highlighted by Gutheil and Gabbard (1993) and Smith and Fitzpatrick (1995), the complexity of care in this field often obscures the boundary between legitimate care and misconduct, posing challenges to detection, reporting, and accountability. This scenario underscores the need for stricter ethical oversight and protective protocols for patients.

An additional concern was that physicians were not always practicing in their certified specialties, yet gynecology and obstetrics remained the most frequently reported field, likely due to the aforementioned factors.

As in previous studies (Cohen et al., 2009; Garrett, 2002; Pope, 1994; State Medical Board of Ohio, 2013), the vast majority of victims were female, underscoring the gendered dimension of sexual abuse in healthcare settings. This reflects longstanding social constructs that position women as subordinate in hierarchical contexts like the physician–patient relationship. Deeply ingrained gender norms—such as delegitimizing female voices and normalizing invasive behavior under the guise of care—facilitate the persistence of abuse. The socialization of women into silence, along with victim-blaming, further perpetuates this pattern and reveals the structural nature of gender inequality in healthcare.

The majority of complaints were initiated by CRM-MG, as observed in previous studies by Cohen et al. (2009) and Marques Filho (2006). This highlights the key role of regulatory bodies in detecting and responding to professional misconduct. Nevertheless, the participation of patients and their families—though smaller—reflects growing social awareness and shared responsibility in addressing such violations.

Private healthcare institutions were most frequently implicated, echoing findings by Gomes (2007), who argued that the discrete environment of private practice, coupled with professional seniority and accumulated socioeconomic power, may foster abusive behavior. However, Cohen et al. (2009) found no significant differences between public and private institutions, suggesting that systemic abuse is not exclusive to any specific healthcare setting.

Regarding the most frequently cited chapters of the Medical Code of Ethics, the chapters on human rights, the physician–patient relationship, and professional responsibility were identified in descending order of prevalence, all of which are closely linked to the respect for human dignity. Concerning the most violated chapters, those relating to the physician–patient relationship, human rights, and professional responsibility stood out, aligning with the provisions most frequently used by board members to classify sexual harassment practices.

The chapter dedicated to “Human Rights” is based on the respect for human dignity and bioethical principles in a clear and objective manner. The provisions of this section address patient autonomy, respect for human dignity, the promotion of non-discrimination, the prohibition of using the profession to alter social values or to commit or facilitate unlawful practices, and the obligation to report practices of torture or degrading, inhuman, or cruel treatment, among others.

The chapter titled “Relationship with Patients and Families” addresses the respect for the autonomy of patients and their legal representatives, incorporating the principles of beneficence and non-maleficence. This normative section emphasizes the prohibition of behaviors that disrespect patient intimacy, as well as the prevention of exploiting the physician–patient relationship for personal gain—whether physical, emotional, financial, or of any other nature.

Among the most violated articles, those directly related to the protection of human dignity stood out. Article 38 of the Medical Code of Ethics, which prohibits physicians from violating the intimacy of those under their care, is directly related to the context of sexual harassment committed by physicians against patients, as it aims to protect the dignity and emotional integrity of patients during medical care. The fundamental principle of this rule is respect for patient autonomy and their physical and psychological integrity—elements essential to the trust in the physician–patient relationship. More broadly, the violation of intimacy can be seen as a form of manipulation or coercion, aimed at satisfying the physician's personal desires at the expense of the patient's dignity. This conduct is, therefore, not only ethically unacceptable but also legally punishable, as it violates professional conduct standards essential to the preservation of medical ethics and patient rights.

Article 40 addresses the abuse of the physician–patient relationship for the personal gain of any kind. This provision reflects an ethically unacceptable behavior in medical practice, characterized by the use of patient trust and vulnerability for selfish purposes, whether physical, emotional, or financial. The transgression of this principle compromises the fundamental trust between the physician and the patient. Sexual harassment by a physician against a patient constitutes a direct violation of this article, as the professional takes advantage of their position of authority and the patient's vulnerability to obtain emotional and sexual gratification.

Article 30 of the Medical Code of Ethics provides a clear normative foundation to regard sexual harassment as a severe ethical violation, going beyond a mere breach of patient respect to constitute the abuse of a professional position for personal and criminal purposes, with harm to both the victim and the profession itself.

Article 23, which establishes the obligation for physicians to treat people with civility, consideration, and respect for dignity, prohibiting any form of discrimination, emphasizes the fundamental principles of respect and dignity that must guide medical conduct in all interactions—especially when attending to patients, whose physical, emotional, and psychological vulnerabilities require impeccable ethical and professional conduct.

The high rate of acquittals observed in this study aligns with previous findings, which also identified a predominance of dismissals or judgments based on the lack of merit of the complaints. This trend largely stems from the evidentiary difficulty inherent in the dynamics of sexual harassment within the medical context, whose intimate and private nature—often restricted to the confines of the consultation room—impedes the acquisition of robust evidence, weakening the accusation and, consequently, the accountability of the defendant (Cohen et al., 2009; Gomes, 2007; Mariani, 2021). In this regard, it becomes imperative for women to be aware of this structural vulnerability and adopt preventive strategies, such as requesting the presence of a companion during intimate medical consultations whenever possible. Such a measure, supported by ethical and legal norms, may not only protect the patient but also ensure greater transparency and security in the physician–patient relationship.

Regarding penalties, when the violation was duly proven, severe sanctions were imposed, in line with the penalties most frequently applied in similar investigations found in specialized literature (Dehlendorf & Wolfe, 1998; Federation of State Medical Boards, 2011). When proven to be unlawful, such violations must be punished with severe and exemplary sanctions, aimed at preventing recidivism and discouraging future abuses, reaffirming the unshakable commitment of medicine to ethical values, professional integrity, and the unconditional protection of patient rights.

It was noted that most of the ethical proceedings involved primary offending physicians, that is, those without prior ethical violations, who had a recidivism rate of 5.4%, a figure similar to a study that identified a recidivism rate of 15.6% (Gomes, 2007). The recidivism of physicians in sexual harassment behavior warrants attention not only from a legal standpoint but also from a clinical one, suggesting the need for a multidisciplinary approach that includes psychological or psychiatric evaluations of offending professionals, both for diagnostic and preventive purposes.

Regarding appeals submitted to the Federal Council of Medicine (CFM), there was a predominance of cases in which no request for review of the first-instance decision by the Regional Medical Council of Minas Gerais (CRM-MG) was made. This scenario might indicate, on one hand, the acceptance by the accused of the penalties imposed, either due to recognition of the legitimacy of the decisions or an assessment that any potential appeals would not substantially alter the outcome. On the other hand, it might reflect a limitation of access or a defensive strategy. In cases where appeals were filed, most resulted in the maintenance of the originally imposed penalty, highlighting that the CFM, acting as the appellate body, tended to preserve the merit judgment made by the CRM, recognizing the regional councils’ proximity and familiarity with the facts, evidence, and local context specifics. This stance reinforced institutional confidence in the ethical-disciplinary process developed by the CRMs, valuing their technical competence and decision-making autonomy.

Concerning associations identified in the study, significant correlations were observed between the chapter on physician–patient relationships and the variable of medical specialty. It was found that specialist physicians had a higher incidence of transgressions related to articles in the physician–patient relationship chapter, compared to other sections of the Medical Code of Ethics. Although it was expected that the more advanced technical and ethical training of specialists would act as a protective factor, the data revealed a higher incidence of ethical violations among these professionals in this domain. This finding raises important hypotheses: the greater autonomy inherent in specialized practice, along with a possible self-perception of consolidated authority, could contribute to a distancing from fundamental ethical principles, favoring behavioral deviations. These aspects require further investigation, especially regarding the interpersonal dynamics established in specialties that involve closer physical or emotional proximity to patients.

Concerning the chapter of the Medical Code of Ethics related to human rights, a significant association was observed between this theme, the type of complainant, and the location of the violation. Specifically, it was found that the CRM-MG was responsible for a higher proportion of complaints related to this chapter, compared to those filed by patients or their families. This finding suggests a proactive institutional role by CRM-MG in identifying behaviors that infringe upon the fundamental rights of patients, signaling a commitment to ethical oversight and the protection of human dignity in medical practice.

Moreover, the analysis revealed that infractions occurring in the interior of the state showed a higher association with violations of human rights compared to those registered in the capital. This pattern could reflect specific structural and cultural characteristics of rural regions, such as lower institutional oversight, barriers to patient access to information, or physician–patient relationships marked by more pronounced power asymmetries. These factors, either individually or collectively, could contribute to an environment more prone to the perpetuation of abusive ethical practices, particularly concerning the violation of dignity and fundamental rights of women in situations of healthcare vulnerability.

A correlation was also observed between specialization and culpability, with non-specialized physicians showing a higher frequency of convictions, contrasted with a lower rate of acquittals. This data points to the need for further investigation into the dynamics involved in this relationship, to better understand the factors contributing to the higher incidence of convictions among general practitioners. Additionally, this analysis could broaden the understanding of how ethical decisions and professional responsibility are influenced by the level of specialization, providing insights for future guidelines for medical training and oversight.

Although this study provides valuable initial information on sexual harassment cases analyzed by CRM-MG, it is important to acknowledge some limitations that may affect the interpretation of the results. Firstly, the limited amount of data available for analysis may restrict the extent of the conclusions. Moreover, the lack of specific bibliographic references on the subject within the context of regional and even international medical boards hinders direct comparison with other similar studies and limits a broader contextualization of the results. Another relevant factor is the probable underreporting of harassment cases, as victims may often feel intimidated or fearful of reporting, either due to the perpetrator's position of power, shame, or fear of professional retaliation. This underreporting could lead to an underestimation of the actual incidence of harassment, compromising the accuracy of the analyzed data.

This is an issue that demands a coordinated and urgent response from regional councils and other entities responsible for oversight, to ensure that medicine is practiced in an ethical, respectful, and abuse-free manner. These restrictions, therefore, highlight the need for future research to expand the data pool and deepen the analysis on the subject, as well as to reinforce the importance of public policies and educational campaigns that encourage reporting and victim protection.

## Conclusion

This study documents concrete evidence of sexual harassment in the physician–patient relationship, based on the analysis of ethical-professional proceedings judged by CRM-MG between 2012 and 2022. The data reveals a scenario of significant underreporting, driven by factors such as fear, shame, and institutional disbelief, which particularly affect adult women in clinical and emotional vulnerability—reinforcing the structural nature of gender violence in this context.

The accused physicians were predominantly male, with an average age of 46 years, extensive professional experience, and mostly without formal specialization. The concentration of cases in gynecology and obstetrics, combined with the predominance of incidents in private institutions in rural areas, highlights the fragility of control mechanisms in environments marked by greater isolation and less institutional oversight. Ethical decisions revealed recurring challenges in producing evidence, leading to frequent acquittals, although penalties applied in proven cases reflected the seriousness of the violations.

The infractions analyzed were primarily framed within ethical provisions concerning the physician–patient relationship and the protection of human rights, in line with the Medical Code of Ethics regarding the repression of behaviors characterized as sexual harassment. This normative alignment underscores the centrality of ethical regulation as an essential tool in confronting inequalities and abuses of power inherent in therapeutic relationships.

The association between medical specialization and higher incidence of infractions in the physician–patient relationship indicated the need for stricter scrutiny regarding the use of professional autonomy and the potential ethical deviations that may arise from it. Furthermore, a significant correlation was observed between the type of complainant and the classification of the violation as a human rights infringement, with CRM-MG playing a central institutional role in this aspect. The fact that rural areas exhibited a higher incidence of human rights violations suggested that more remote regional contexts may require specific educational interventions.

The discussion surrounding sexual harassment in medicine constitutes an ethical, political, and scientific imperative, demanding not only visibility of victims and the harm caused by such conduct but also a critical exposure of the structures of domination and the mechanisms of silence that persist in clinical practice.

The production and dissemination of empirical studies, such as the one presented here, are crucial for advancing scientific knowledge, improving institutional norms, and strengthening professional accountability and prevention mechanisms. In this regard, the findings of this investigation reiterate the urgency of implementing concrete preventive measures, such as awareness campaigns, widespread dissemination of information on ethical conduct in clinical contexts, and the effective implementation of patients’ rights to have a companion present during consultations involving body exposure or potential embarrassment.

Moreover, it is imperative that professional boards and educational institutions commit to the continuous promotion of ethical training and the creation of effective accountability mechanisms, in order to break the silence culture that still pervades the healthcare field and ensure physician–patient relationships based on human dignity, respect, and equity.

## Annex 1. Section of the Code of Medical Ethics—Resolution CFM n_0_. 2,217, of September 27, 2018, as Amended by Resolutions CFM n_0s_. 2,222/2018 and 2,226/2019—Involving the Most Frequently Cited and Violated Chapters in Cases of Sexual Harassment in Medical Practice in a Brazilian State.

**Table:**

*Chapter III—Professional Responsibility* *Article 1.* It is prohibited for the physician to cause harm to the patient through action or omission, characterized as lack of skill, negligence, or imprudence. *Sole paragraph.* Medical responsibility is always personal and cannot be presumed. *Article 2.* It is prohibited for the physician to delegate to other professionals acts or assignments that are exclusive to the medical profession. *Article 3.* It is prohibited for the physician to fail to assume responsibility for a medical procedure they indicated or participated in, even when multiple physicians attended to the patient. *Article 4.* It is prohibited for the physician to fail to assume responsibility for any professional act they performed or indicated, even if requested or consented to by the patient or their legal representative. *Article 5.* It is prohibited for the physician to assume responsibility for a medical act they did not perform or participate in. *Article 6.* It is prohibited for the physician to attribute their failures to third parties or to circumstantial factors, except in cases where this can be duly proven. *Article 7.* It is prohibited for the physician to fail to attend to urgent and emergency sectors when required to do so, even when supported by a majority decision of the profession. *Article 8.* It is prohibited for the physician to withdraw from their professional activities, even temporarily, without assigning another physician to be in charge of attending to their hospitalized or critically ill patients. *Article 9.* It is prohibited for the physician to fail to show up for a shift at a pre-established time or to abandon their shift without a substitute, except in the case of a just impediment. *Sole paragraph.* In the absence of a substitute physician, the technical management of the healthcare facility must provide for the substitution. *Article 10.* It is prohibited for the physician to collude with individuals who practice medicine illegally or with professionals or medical institutions where illicit acts are carried out. *Article 11.* It is prohibited for the physician to prescribe, attest, or issue reports in a secret or illegible manner, without proper identification of their registration number in the Regional Medical Council of their jurisdiction, or to sign blank prescription sheets, certificates, reports, or any other medical documents. *Article 12.* It is prohibited for the physician to fail to inform the worker about working conditions that may jeopardize their health, and the physician must report the matter to the responsible employers. *Sole paragraph.* If the situation persists, it is the physician's duty to report the occurrence to the competent authorities and the Regional Medical Council. *Article 13.* It is prohibited for the physician to fail to inform the patient about the social, environmental, or professional determinants of their illness. *Article 14.* It is prohibited for the physician to perform or recommend unnecessary or prohibited medical acts, according to the current legislation in the country. *Article 15.* It is prohibited for the physician to violate specific legislation related to organ or tissue transplants, sterilization, artificial insemination, abortion, or genetic manipulation or therapy. *§ 1.* In the case of medically assisted procreation, fertilization should not systematically result in surplus embryos.	*§ 2.* The physician must not engage in medically assisted procreation with any of the following goals: I.To create genetically modified human beings; II.To create embryos for research purposes; III.To create embryos for sex selection, eugenics, or to generate hybrids or chimeras. *§ 3.* The physician must not perform medically assisted procreation procedures unless all participants are fully in agreement and adequately informed about the method. *Article 16.* It is prohibited for the physician to intervene in the human genome for modification purposes, except in gene therapy, excluding any action on germ cells that results in genetic modification of descendants. *Article 17.* It is prohibited for the physician to fail to comply, without a valid reason, with the regulations issued by the Federal and Regional Medical Councils, or to fail to respond to their administrative requests, summonses, or notifications within the determined period. *Article 18.* It is prohibited for the physician to disobey or disrespect the decisions and resolutions of the Federal and Regional Medical Councils. *Article 19.* It is prohibited for the physician to fail to ensure, when in a position of leadership, the rights of physicians and the necessary conditions for the ethical-professional practice of medicine. *Article 20.* It is prohibited for the physician to allow financial, political, religious, or any other interests of their employer, superior, or public or private healthcare funding entity to interfere with the choice of the best available scientifically recognized methods of prevention, diagnosis, or treatment for the patient's or society's health. *Article 21.* It is prohibited for the physician to fail to cooperate with health authorities or to infringe relevant legislation. *Chapter IV – Human Rights* *Article 22.* It is prohibited for the physician to fail to obtain the patient's or their legal representative's consent after providing adequate information about the procedure to be performed, except in cases of imminent risk of death. *Article 23.* It is prohibited for the physician to treat any human being without civility or consideration, to disrespect their dignity, or to discriminate against them in any way or under any pretext. *Sole paragraph.* The physician must maintain respect, consideration, and solidarity with their colleagues. *Article 24.* It is prohibited for the physician to fail to guarantee the patient's right to freely decide about their own person or well-being, or to exercise their authority to limit such rights. *Article 25.* It is prohibited for the physician to fail to report the practice of torture or degrading, inhuman, or cruel procedures, to engage in such practices, to condone those who perform them, or to provide means, instruments, substances, or knowledge that facilitate such acts. *Article 26.* It is prohibited for the physician to fail to respect the will of any individual who is considered physically and mentally capable, in a state of hunger strike, or to force-feed them, without adequately informing them of the probable (continued)	complications of prolonged fasting. In cases of imminent risk of death, treatment must be provided. *Article 27.* It is prohibited for the physician to disrespect the physical and mental integrity of the patient or to use methods that may alter their personality or consciousness in police investigations or any other type of inquiry. *Article 28.* It is prohibited for the physician to disregard the interest and integrity of the patient in any institution where they are confined, irrespective of the patient's own will. Sole paragraph. In the event of any acts that harm the personality or the physical or mental health of patients under the physician's care, the physician is obligated to report the matter to the competent authority and the Regional Medical Council. *Article 29.* It is prohibited for the physician to participate, directly or indirectly, in the execution of the death penalty. *Article 30.* It is prohibited for the physician to use their profession to corrupt morals, commit, or facilitate crimes. *Chapter V – Relationship with Patients and Families* It is prohibited for the physician to: *Article 31.* Disregard the patient's or their legal representative's right to freely decide on the execution of diagnostic or therapeutic practices, except in cases of imminent risk of death. *Article 32.* Fail to use all available means of health promotion and disease prevention, diagnosis, and treatment, scientifically recognized and within their reach, in favor of the patient. Article 33. Fail to attend to a patient seeking professional care in cases of urgency or emergency when no other physician or medical service is available to do so. *Article 34.* Fail to inform the patient of the diagnosis, prognosis, risks, and objectives of the treatment, except when direct communication may cause harm; in such cases, the information should be communicated to the legal representative. *Article 35.* Exaggerate the severity of the diagnosis or prognosis, complicate the therapeutic process, or exceed the number of visits, consultations, or any other medical procedures. *Article 36.* Abandon a patient under their care. *§ 1°.* If circumstances arise that, in the physician's judgment, harm the good relationship with the patient or full professional performance, the physician has the right to withdraw from the treatment, provided they communicate this in advance to the patient or their legal representative, ensuring continuity of care and providing all necessary information to the succeeding physician. *§ 2°.* Unless for just cause communicated to the patient or their family, the physician shall not abandon the patient due to chronic or incurable illness and shall continue to assist them, including providing necessary palliative care.	*Article 37.* Prescribe treatment or other procedures without direct examination of the patient, except in cases of urgency or emergency and when it is impossible to perform the examination; in such cases, the examination should be conducted immediately after the impediment is resolved. *Sole paragraph.* Remote medical care, such as telemedicine or other methods, shall be conducted under the regulation of the Federal Medical Council. *Article 38.* Disregard the modesty of any person under their professional care. *Article 39.* Oppose the performance of a medical board or second opinion requested by the patient or their legal representative.​ *Article 40.* Exploit situations arising from the physician–patient relationship to gain physical, emotional, financial, or any other advantage. *Article 41.* Shorten the patient's life, even at their or their legal representative's request. *Sole paragraph.* In cases of incurable and terminal illness, the physician must offer all available palliative care without undertaking futile or obstinate diagnostic or therapeutic actions, always considering the expressed will of the patient or, in their inability, that of their legal representative. *Article 42.* Disregard the patient's right to freely decide on a contraceptive method, always providing information on the indication, safety, reversibility, and risks of each method.
